# Evaluation of forage production performance and prediction of introduction adaptability for different *Avena sativa* germplasms in the southeast edge of the Qinghai-Tibet plateau

**DOI:** 10.3389/fpls.2025.1564278

**Published:** 2025-08-13

**Authors:** Ting Zhao, Huanhuan Lu, Fan Zhang, Yuying Zheng, Liuban Tang, Changbing Zhang, Yongcai Ma, Rongjia Cai, Wengang Xie

**Affiliations:** ^1^ State Key Laboratory of Herbage Improvement and Grassland Agro-Ecosystems, Ministry of Agriculture and Rural Affairs, College of Pastoral Agriculture Science and Technology, Lanzhou University, Lanzhou, China; ^2^ Key Laboratory of Grassland Livestock Industry Innovation, Ministry of Agriculture and Rural Affairs, College of Pastoral Agriculture Science and Technology, Lanzhou University, Lanzhou, China; ^3^ Sichuan Academy of Grassland Science, Chengdu, China; ^4^ Livestock Comprehensive Experimental Field of Gannan Tibetan Autonomous Prefecture, Hezuo, China

**Keywords:** *Avena sativa*, forage production performance, Qinghai Tibet plateau, distribution of potential suitability regions, prediction of introduction adaptability

## Abstract

**Introduction:**

The grassland degradation and harsh climate in the Qinghai-Tibet Plateau (QTP) have constrained forage production, thereby impeding the development of animal husbandry in pastoral areas of China. The effective cultivation of high-yielding *Avena sativa* could help alleviate this constraint.

**Methods:**

This study comprehensively evaluated the forage yield and related agronomic traits of 62 *A. sativa* germplasms. Additionally, the introducing adaptability of different germplasms was predicted across various regions of the QTP.

**Results:**

The results indicated that: (1) 43 of 62 germplasms could successfully completed the fertility period in the eastern edge of the QTP; (2) Fresh and hay grass yield were significantly positively correlated with absolute plant height, flag leaf length, flag leaf width, flag leaf area, flag-second leaf length, flag-second leaf width, flag-second leaf area, number of total tiller, number of vegetative tiller, stem to leaf ratio of hay, thick of second stem internode, spike length, and root depth, while significantly negatively correlated with thousand kernel weight; (3) Fresh and hay grass yield were directly affected by flag-second leaf length, flag-second leaf width, flag-second leaf area, number of total tiller, number of fertile tiller, and stem to leaf ratio of hay; (4) *A. sativa* germplasm AS55 exhibited the highest comprehensive forage performance under field conditions, followed by AS39, AS48, AS28, AS41, and AS20; (5) The potential spatial distribution of *A. sativa* was concentrated in the southeastern QTP, with its distribution being significantly affected by hydrothermal conditions. Moreover, the potential distribution pattern of high suitability regions showed an expanding trend over time; (6) 15 *A. sativa* germplasms had varying degrees of introduction adaptability in 23 municipal-level administrative regions within the QTP. The potential suitable area for introducing different germplasms was 7.81×10^5^ km^2^ in the current period and 2.68×10^5^ km^2^ in the future period. the area of regions with high introduction adaptability increased from 1.60×10^3^ km^2^ in the current period to 1.38×10^4^ km^3^ in the future period.

**Discussion:**

In this study, evaluations of forage production performance and predictions of introduction adaptability were conducted for 62 *A. sativa* germplasms at the eastern edge of the QTP. The findings aim to provide a theoretical basis for the collection, preservation, and utilization of *A. sativa* germplasm resources, as well as valuable insights for the cultivation and protection of plants in the QTP.

## 1 Introduction

The Qinghai-Tibet Plateau (QTP), located in southwestern China with an average elevation exceeding 4,000 m, is the highest and largest plateau globally. It not only encompasses the world’s largest natural alpine grassland but also serves as China’s most critical pastoral area (Cao et al., 2018; Zhou et al., 2023). However, the current development of animal husbandry in this region faces multiple challenges, including grassland degradation, insufficient forage supply, and the imbalance between grass resources and livestock (Li et al., 2020). Especially, the shortage of forage resulting from the prolonged winter-spring hay period on the QTP has emerged as a major obstacle restricting the development of animal husbandry and even the social economy (Dong et al., 2011). Sown pastures can effectively address the spatiotemporal mismatch between forage production and animal husbandry development, which is of great significance for alleviating grassland degradation, improving the ecological environment, maintaining ecological balance, and enhancing the output value of animal husbandry (Liu et al., 2023). Due to low temperatures, a short growing season, and climate instability, forage yield and quality in the QTP region are relatively low (Zhang et al., 2015). Therefore, breeding high-yield and high-quality forage represents an effective measure to increase cold-season forage supply, thereby promoting the sustainable development of animal husbandry in alpine regions.

As an annual herbaceous plant belonging to the Poaceae family, *Avena sativa* serves not only as a human food source but also as a forage, owing to its succulent stems and leaves, high crude protein content, and balanced amino acid composition (Shah et al., 2015). As a forage, *A. sativa* exhibits advantages such as cold tolerance, salt-alkali tolerance, strong adaptability, and high yield, making it one of the most widely used high-quality forages in sown pasture establishment (Ye et al., 2023). According to data from the Food and Agriculture Organization of the United Nations (FAO, https://www.fao.org/faostat/zh/#data/QCL, accessed 24 November 2024), the planting area of *A. sativa* in China amounted to approximately 7.70×10^5^ hm^2^ by 2021, with a grain yield of 6.52×10^5^ tons. Among this, the planting area of *A. sativa* for commercial forage exceeded 1.70×10^5^ hm^2^, producing over 1.30×10^6^ tons of hay. When planting for the purpose of forage yield, except for considering the intuitive evaluation indicators brought by the biomass, it is also necessary to evaluate the growth performance of *A. sativa* by analyzing agronomic traits closely related to forage yield (Wang et al., 2019) so as to more effectively distinguish the regional adaptability of target germplasm. Furthermore, germplasms in different regions are constrained by environmental conditions, climate, and cultivation practices, leading to variations in the production performance of *A. sativa* across regions (Sánchez-Martín et al., 2014). Conversely, different germplasms exhibit distinct adaptability and growth potential due to differences in heritability (Kebede et al., 2023). Therefore, the evaluation of forage yield and adaptability not only constitutes a key step in selecting elite A. sativa germplasms but also lays the foundation for identifying characteristic traits of genotypes with high forage yield potential.

Species distribution models (SDMs) are widely used to predict the potential geographical distribution of species, assess ecological protection, and analyze the characteristics of species-suitable regions (Miller, 2010). Ensemble species distribution models (ESDMs) integrate multiple SDMs to eliminate biases and uncertainties among different algorithms, thereby generating more robust results and insights (Lu et al., 2025). Predicting introduction adaptability based on environmental similarity can effectively reduce management costs and facilitate the construction of a priority network for germplasm-suitable regions (Steinitz et al., 2006; Watts et al., 2017). To effectively screen high-yield and high-quality *A. sativa* germplasms suitable for cultivation on the QTP, this study performed descriptive analysis, grey relational analysis, path analysis, membership function analysis, and introduction adaptability prediction on the forage yield and related traits of 62 germplasms. The objective was to answer three scientific questions: (1) What are the production performance characteristics of different *A. sativa* germplasms? (2) What are the potential spatiotemporal distribution patterns of *A. sativa* in the QTP region? (3) What are the regional adaptation characteristics of different *A. sativa* germplasms introduced to the QTP region? The high-yield *A. sativa* germplasms selected in this study are expected to play an important role in sustaining the development of grassland animal husbandry in the alpine pastoral regions of the QTP.

## 2 Materials and methods

### 2.1 Analysis of potential spatiotemporal distribution pattern of *A. sativa* in the QTP region

Due to the lack of distribution points in the QTP region for modeling, the global distribution points of *A. sativa* were comprehensively compiled from the existing literature, field investigation and public online database. Four online databases (GBIF, Ecoengine, iNaturalist and iDigBio) related to plant distribution information were queried by the R package spocc v1.2.2 (https://cran.R-project.org/package=spocc), and the search ended on October 1, 2024. The distribution data were checked for the common spatial and temporal errors using the R package CoordinateCleaner v3.0.1 (Zizka et al., 2019). To reduce spatial autocorrelation and sampling bias, the Spatially Rare Occurrence Data tool in SDM Toolbox v2.4 (Brown et al., 2017) was used to rarefy the species distribution data at 5 arc-minutes (~10 km) resolution. This study obtained environmental variables that widely affected the plant growth and distribution of *A. sativa* (**Supplementary Table S1**), including 19 sets of bioclimatic factors and 1 set of topographic factors. Bioclimatic data were sourced from Paleoclim website (http://www.paleoclim.org). Current bioclimate (CP, 1979~2013) were obtained using Anthropocene v1.2b dataset, while the future bioclimate (FP, 2071~2100) were obtained using CMIP6 dataset under the MPI-ESM1-2-HR climate model (Jägermeyr et al., 2021) and the ssp375 economic sharing pathway (Riahi et al., 2017). These bioclimatic variables mainly represent long-term annual trends, seasonality, and extreme situations of temperature and precipitation. Elevation indirectly affects the adaptability of species to the environment by affecting temperature, and the SRTM elevation data was selected from the WorldClim website (https://www.worldclim.org). All environmental variables were unified into GCS_WGS_1984, resampled to 5 arc-minutes resolution, and masked to global regions by ArcGIS v10.8 (https://desktop.arcgis.com/). Global regions were created from the map with review number GS (2016) 1663, and the base map was not modified. To avoid the multicollinearity of environmental variables leading to over-fitting of prediction model, the Correlations and Summary Stats tool in SDM Toolbox software was used to detect the correlation among environmental variables, and retaining only environmental variables with |*r*| < 0.80 and significant ecological significance.

The potential spatial distribution pattern of *A. sativa* in the current and future periods was modeled and predicted by R package ssdm v0.2.9 (Schmitt et al., 2017). To obtain more robust prediction results, ESDMs method in ssdm software was selected, which included 9 modeling algorithms, such as Maximum Entropy (MAXENT), Generalized Linear Model (GLM), Generalized Additive Model (GAM), Multiple Adaptive Regression Splines (MARS), Generalized Boosting Model (GBM), Classification Tree Analysis (CTA), Random Forest (RF), and Artificial Neural Network (ANN) and Support Vector Machine (SVM). During the modeling process, the pseudo-occurrence points of the species were automatically generated, and 75% distribution data were randomly divided into training datasets for model construction and the remaining were used as testing dataset to evaluate model performance. The independence between training and testing datasets was verified by 10 repeated crossover method based on holdout strategy. The area under the curve of receiver operating characteristics (AUC) threshold is 0.75 as the standard for ensemble modeling algorithms, and pearson correlation tests were performed among algorithms. An integrated scoring system of TSS, Kappa and AUC were used to evaluate the predictive performance of the ESDMs method (Lu et al., 2025). The relative importance of environmental variables was estimated by the ssdm software, and the ssdm software generated continuity distribution probability with a threshold accuracy of 100. It is worth noting that this study predicted the global distribution probability of *A. sativa*, and then masked them to the QTP region. The QTP region was created from the map with review number GS (2019) 1822.

To further evaluate the spatial distribution patterns of *A. sativa* at different periods, based on the natural break classification method, the distribution probability was divided into three suitability levels (high-, low-, and un-suitability regions) by using the Reclassify tool in ArcGIS software, and the area of different suitability levels in each administrative region was counted by using the Region analysis tool in ArcGIS software. In addition, the change and migration of suitability regions are a response to environmental changes over a long period of time. The geometric centroid of suitability regions is used as a general description of the distribution characteristics of species in each period, and the migration of geometric centroid can more intuitively display the changes of suitability regions in different periods. To explore the temporal distribution pattern of *A. sativa* from the current (1979~2013) to future (2071~2100) period, the geometric centroid positions in different periods were located by the Region analysis tool in ArcGIS software and R package foreign v0.8 (https://cran.r-project.org/package=foreign), and the geographical distance between different centroids was calculated by R package geosphere v1.5 (https://cran.r-project.org/package=geosphere).

### 2.2 Germplasms sources, experimental region and field cultivation measures

According to the potential suitability distribution pattern of *A. sativa*, the experimental region was selected in the southeast edge of the QTP. The region belonged to a typical continental plateau cold temperate monsoon climate, with annual average temperature of 0.91 °C, annual temperature range of 16.3 °C, annual precipitation of 650~800 mm, net primary productivity of 691.03 gC·m^-2^·yr^-1^, no absolute frost-free period, distancing dry and rainy season, and low heat. The soil pH value was 7.24, soil organic matter content was 0.32 g·kg^-1^, available phosphorus was 6.19 mg·kg^-1^ and available potassium was 76.42 mg·kg^-1^ at the experimental region.

The present study included 62 A*. sativa* germplasm resources, which were provided by the Institute of Animal Sciences, Chinese Academy of Agricultural Sciences (**Supplementary Table S2**). A one-year field trial was initiated in June 2023 at the Forage Breeding Base of the Grassland Science Research Institute, located in Hongyuan County, Sichuan Province (geographical coordinates: 32°47’N, 102°32’E; altitude: 3460 m). The experiment adopted a randomized block design with 3 replicates, with a sample plot area of 15 m^2^ (3 m × 5 m) and a spacing of 0.5 m between plots. Each sample plot was evenly seeded with 140 g of seeds, the row spacing of 0.3 m and a sowing depth of 5~8 cm. 20 days before sowing, the sample plot was plowed (12~20cm), raked, and not irrigated and fertilized. The weeds were manually removed during the seedling stage.

### 2.3 Determination and analysis of forage yield and related agronomic traits

The forage yield and relative feeding value of *A. sativa* in the filling stage have significant advantages (Wang et al., 2019). To effectively evaluate the production performance and maximize feeding value, 23 agronomic trait indicators for 62 A*. sativa* germplasms were measured and calculated during the filling and maturing stages according to “Description Standard and Data Standard of Oat Germplasm Resources” (Ren et al., 2023), with each indicator repeated 10 times (**Supplementary Table S3**). It is worth noting that after 50% of the plants in each sample plot reached the filling stage, a total of 10 individual plants at the filling stage were randomly selected from the middle of the 3 repeated plots of each germplasm for the determination of fresh grass yield per plant. The fresh individual plants were killed the green at 105 °C for 30 minutes, then dried at 80 °C for 48 hours until constant weight, and hay grass yield per plant was measured. The yield of hay stem/leaf was measured by separating stems and leaves after determining the hay grass yield. In addition, the leaf area was calculated based on the correction coefficient *R*=0.8317 (Liang et al., 2018). To comprehensively describe the agronomic traits of different germplasms, descriptive and variant analysis of different germplasms and agronomic traits was calculated through SPSS v25.0 (https://www.ibm.com/spss). The *post hoc* multiple tests on agronomic traits were tested by online SPSSPRO software (https://www.spsspro.com/) with Tamhane’s T2 strategy. To eliminate the influence of dimensionality, the values of all agronomic traits were normalized by R package vegan v2.6 (https://CRAN.R-project.org/package=vegan). The shannon diversity of different germplasms for describing trait diversity was calculated using the vegan software. The correlation analysis among agronomic traits was calculated using SPSS software with two tailed for significance. Grey relational analysis is a comprehensive method for describing and quantifying the impact of various factors. After weighting different agronomic traits by the entropy weight method, the grey correlation analysis of forage yield and its constituent factors was conducted using SPSSPRO software with the objectives of hay and fresh grass yield, and the resolution coefficient ρ of 0.5. Due to the collinearity between forage yield and its constituent factors, path analysis of hay and fresh grass yield based on ridge regression was calculated using SPSSPRO software to explore the potential contribution of agronomic trait factors to forage yield in detail. To effectively evaluate the differences in forage yield among different germplasms, cluster analysis was performed using vegan software. Principal component analysis (PAC) and phenotypic contribution analysis for agronomic traits were carried out using R package FactoMineR v2.11 (https://CRAN.R-project.org/package=FactoMineR). Based on the value of agronomic traits with contribution ≥ 1, the membership function analysis was carried out by SPSSPRO software to evaluate the comprehensive performance of different *A. sativa* germplasms.

### 2.4 Prediction of introduction adaptability of different *Avena sativa* germplasms in the QTP region

Based on the potential distribution pattern of *A. sativa*, the suitability probability was extracted by using Extraction analysis tool in ArcGIS software under constraints of different municipal administrative in the QTP region. The distribution probability was used as the geographical environment standard to measure the suitable distribution of *A. sativa*, and the agronomic traits were weighted by the entropy weight method to measure the adaptability level of different germplasms. The optimal dissimilarity index was screened by vegan software, and mantel test between municipal administrative regions and weighted agronomic trait values of germplasms was carried out by ChiPlot online software (https://www.chiplot.online/) to predict the introduction adaptability of different germplasms.

## 3 Results

### 3.1 Descriptive evaluation of different *A. sativa* germplasms

19 of 62 A*. sativa* germplasms did not effectively complete fertility period (**Supplementary Table S2**), and the agronomic traits of the remaining germplasms were used for subsequent analysis. The descriptive and variation analysis results of different agronomic traits showed that the coefficient of variation of different agronomic traits ranged from 9.72% to 59.29% (**Table 1**), with a fresh weight of 416.69 ± 107.93g per plant (C.V. of 25.90%) and a dry weight of 122.39 ± 31.23g per plant (C.V. of 25.51%). The descriptive and variation analysis results of different germplasms showed that the average coefficient of variation of different agronomic traits of each germplasm ranged from 10.36% to 34.74% (**Supplementary Table S4**). The variation range of Hay grass yield (HY) of different germplasms was from 74.60 to 197.60 g. Among them, the germplasms with higher yield were AS55 and AS39, both higher than 170 g per plant, and significantly different from AS01, AS02, AS13, AS21, AS40 and AS50 (*p* < 0.05) (**Supplementary Figure S1**). The variation range of Fresh grass yield (FY) ranged from 228.00 g to 707.60 g. The best germplasm was AS55, which was significantly different from AS01, AS02, AS13, AS21, AS46, AS50 and AS61 (*p* < 0.05) (**Supplementary Figure S1**). The yield of hay and fresh grass per plant showed that AS55 germplasm performed the best. In addition, the shannon diversity of different germplasms ranged from 10.49 to 21.52 (**Supplementary Table S5**), indicated that different degrees of phenotypic diversity were helpful for the breeding of different varieties for specific purposes.

**Table 1:** Descriptive and variation analysis of different agronomic traits of *Avena sativa*.

Agronomic traits	Minimum value	Maximum value	Average value	Std.	C.V.
HFR (number)	0.22	0.36	0.30	0.03	9.72%
PH (cm)	102.80	157.80	133.38	12.98	9.73%
RD (cm)	7.50	12.17	9.92	1.10	11.10%
SL (mm)	14.11	21.33	16.83	1.90	11.31%
RL (layer/plant)	5.20	9.20	7.47	0.88	11.78%
SW (mm)	2.45	4.13	3.12	0.42	13.48%
LLf (cm)	21.82	42.90	29.00	4.44	15.30%
SLR (number)	1.20	2.17	1.51	0.25	16.24%
LWf (cm)	1.54	3.60	2.62	0.45	17.06%
TSI (mm)	4.08	9.50	6.27	1.07	17.11%
LLfs (cm)	24.50	63.10	39.45	7.03	17.81%
LWfs (cm)	1.44	3.60	2.60	0.48	18.46%
SPL (cm)	18.36	49.40	30.80	6.63	21.51%
HYL (g)	29.80	73.80	48.52	10.90	22.47%
TKW (g)	13.93	41.54	25.25	6.06	24.00%
HY (g/plant)	74.60	197.60	122.39	31.23	25.51%
FY (g/plant)	228.00	707.60	416.69	107.93	25.90%
LAf (cm^2^)	28.89	129.44	64.79	18.16	28.03%
HYS (g)	43.00	127.20	73.87	21.62	29.27%
NTT (number/plant)	11.80	36.80	20.15	6.10	30.24%
LAfs (cm^2^)	34.06	172.53	87.82	28.86	32.86%
NFT (number/plant)	3.00	24.40	11.51	5.36	46.56%
NVT (number/plant)	1.40	19.00	8.64	5.13	59.29%

Std., standard deviation; C.V., Coefficient of variation; The meanings of abbreviations for agronomic traits were shown in **Supplementary Table S3**.

### 3.2 Correlation analysis of forage yield and its constituent factors

To effectively explore the relationship between forage yield and its constituent factors, the pearson correlation results showed (**Table 2**) that FY was significantly correlated with 16 sets of agronomic trait factors (*p*<0.05), which was significantly positively correlated with remaining agronomic trait factors excepted for thousand kernel weight (TKW) (*r*>0.000, *p*<0.05). There was a significant correlation between HY and 18 agronomic trait factors (*p*<0.05), which was similar to that of FY. The difference was that HY and seed width (SW) had a significant negative correlation (*r*=-0.308, *p*<0.01). The increase of TKW can inhibit the accumulation of HY and FY of *A. sativa*. The grey relational results (**Table 2**) showed that, with a relational degree > 0.75, FY was mainly related to hay stem yield (HYS), hay leaf yield (HYL), flag leaf area (LAf), flag-second leaf length (LLfs), flag-second leaf length (LLf), flag-second leaf area (LAfs), number of vegetative tiller (NVT), spike length (SPL) and thick of second stem internode (TSI); HY was related to HYS, HYL, LLf, LAf, LLfs and LAfs. Overall, HY and FY were both related to leaf biomass. However, FY was also related to stem biomass, which was an important component of plant nutrition and water transport, and can effectively increase plant fresh weight.

**Table 2:** Correlation analysis of forage yield and its constituent factors of *Avena sativa*.

Factor	Dependent variable (HY)			Dependent variable (FY)		
	Correlation	Relevance	Rank	Correlation	Relevance	Rank
HYS	0.980**	0.922	1	0.937**	0.875	1
HYL	0.920**	0.854	2	0.824**	0.829	2
LLf	0.547**	0.784	3	0.637**	0.801	5
LAf	0.611**	0.784	4	0.686**	0.811	3
LLfs	0.617**	0.783	5	0.657**	0.806	4
LAfs	0.621**	0.779	6	0.654**	0.800	6
SPL	0.473**	0.745	7	0.498**	0.768	8
NVT	0.545**	0.743	8	0.571**	0.773	7
SLR	0.526**	0.737	9	0.578**	0.739	11
LWf	0.517**	0.729	10	0.553**	0.746	10
NTT	0.324*	0.722	11	0.303*	0.728	13
LWfs	0.546**	0.715	12	0.563**	0.735	12
TSI	0.304*	0.715	13	0.345*	0.752	9
HFR	0.057	0.688	14	-0.280	0.664	20
RD	0.329*	0.678	15	0.314*	0.701	14
NFT	-0.153	0.669	16	-0.201	0.684	17
PH	0.354*	0.668	17	0.313*	0.671	18
SL	0.057	0.668	18	0.032	0.689	15
RL	0.196	0.655	19	0.246	0.685	16
TKW	-0.610**	0.625	20	-0.547**	0.668	19
SW	-0.308*	0.618	21	-0.197	0.654	21
HY	1.000	—	—	—	—	—
FY	0.936**	—	—	1.000	—	—

“*” and “**” represent significance at the 0.05 and 0.01 levels, respectively; The meanings of abbreviations for agronomic traits were shown in **Supplementary Table S3**.

### 3.3 Path analysis of forage yield and its constituent factors

To further explore the effects of different agronomic traits on forage yield, the path analysis showed that LLf, LLfs, Flag-second leaf width (LWfs), LAfs, number of total tiller (NTT), number of fern tiller (NFT) and stem to leaf ratio of hay (SLR) can directly affect the FY (**Supplementary Table S6**); while HY was directly impacted by FY, hay to fresh ratio (HFR), LLfs, LWfs, LAfs, NTT, NNFT, HYS, HYL and SLR (**Supplementary Table S7**). The top three agronomic traits that directly affect FY and HY of *A. sativa* separately were NTT, NFT, SLR, and HYL, HYS, FY. Overall, compared to leaf traits (flag and flag-second leaf), stem traits have a greater contribution to the accumulation of FY; HY was more influenced by the dry leaf-stem yield on the basis of the accumulation of FY. This difference highlights the difficulty of selecting that higher FY may not necessarily result in higher HY. Therefore, when taking FY and HY as breeding objectives, it is necessary to consider the importance of different agricultural traits.

### 3.4 Comprehensive evaluation of different germplasms and its forage yield

**Figure 1:**
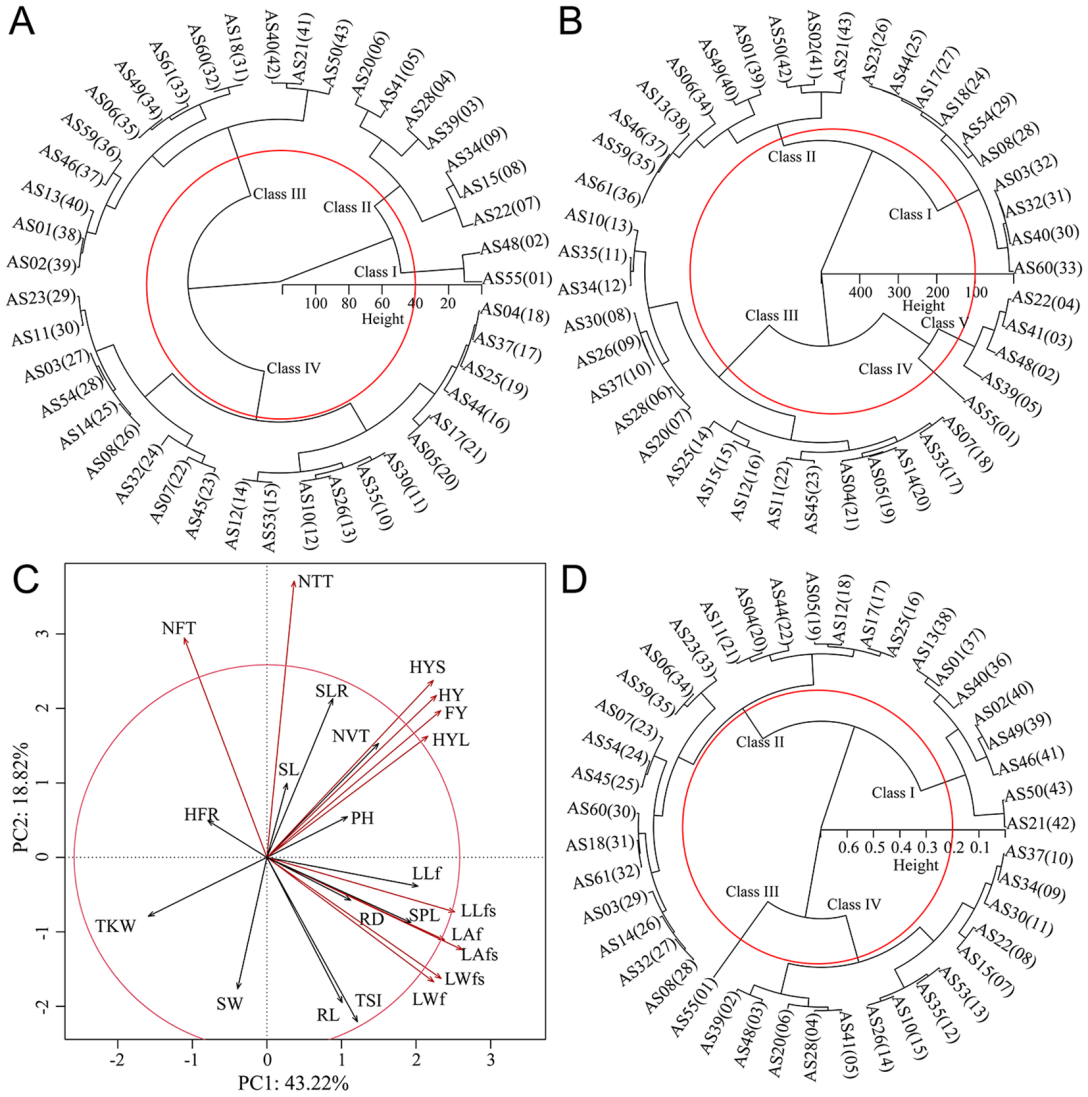
Forage yield and its comprehensive evaluation of*A. sativa*. **(A, B)** Cluster analysis of hay **(A)** and fresh grass **(B)** yield of*A. sativa*; **(C)** PCA and contribution analysis of agronomic traits; **(D)** Cluster analysis of comprehensive evaluation of*A. sativa* based on membership function values. The meanings of abbreviations for germplasms and agronomic traits were shown in **Supplementary Tables S2** and **S3**, respectively.

To individual evaluate the quality of different germplasms on FY and HY, Cluster analysis showed that when the Euclidean distance of HY was 40, different germplasms can be divided into four classes (**Figure 1A**). Class I only included two germplasms AS55 (197.60 g) and AS48 (187.00 g), which had the highest grass yield; HY was closely followed by Class II, that included AS39, AS28, AS41, AS22, AS20, AS15 and AS34, with HY ranged of 148.60 to 176.00 g. When the Euclidean distance of FY was 100, germplasms can be divided into 5 classes (**Figure 1B**). Class IV included one germplasm AS55, which had the highest grass yield; Class V included four germplasms AS48, AS41, AS22 and AS39, which had high grass yields ranging from 554.00 to 606.20 g.

To comprehensively evaluate the quality of different germplasms, the PCA and contribution analysis showed that all agronomic traits explained 62.04% of the overall traits (**Figure 1C**), among which 11 agronomic traits, such as HY, FY, LWf, LAf, LLfs, LWfs, LAfs, NTT, NFT, HYS and HYL, contributed significantly to the comprehensive growth evaluation of *A. sativa*. The membership function values for different germplasms ranged 0.14 to 0.82. The clustering analysis of membership function values (**Figure 1D**) showed that at Euclidean distance of 0.2, different germplasms were divided into four classes: Class III included AS55 germplasm, with a membership function value of 0.82, indicating that AS55 had the highest comprehensive field production performance; Class IV included 10 germplasms with membership function values ranging from 0.48 to 0.64, both hay and fresh grass yields were higher, indicating that germplasms in Class IV had higher comprehensive field production performance; Class II included 20 germplasms with membership function values ranging from 0.27 to 0.43, the germplasms of this class generally showed good seed yield and shorter plant height; Class I included 8 germplasms with membership function values ranging from 0.14 to 0.26, of germplasms had lower field production performance. Overall, AS55 had the best field production performance, followed by AS10, AS15, AS20, AS22, AS26, AS28, AS30, AS34, AS35, AS37, AS39, AS41, AS48 and AS53.

### 3.5 Potential spatiotemporal distribution pattern of *A. sativa* in the QTP region

**Figure 2:**
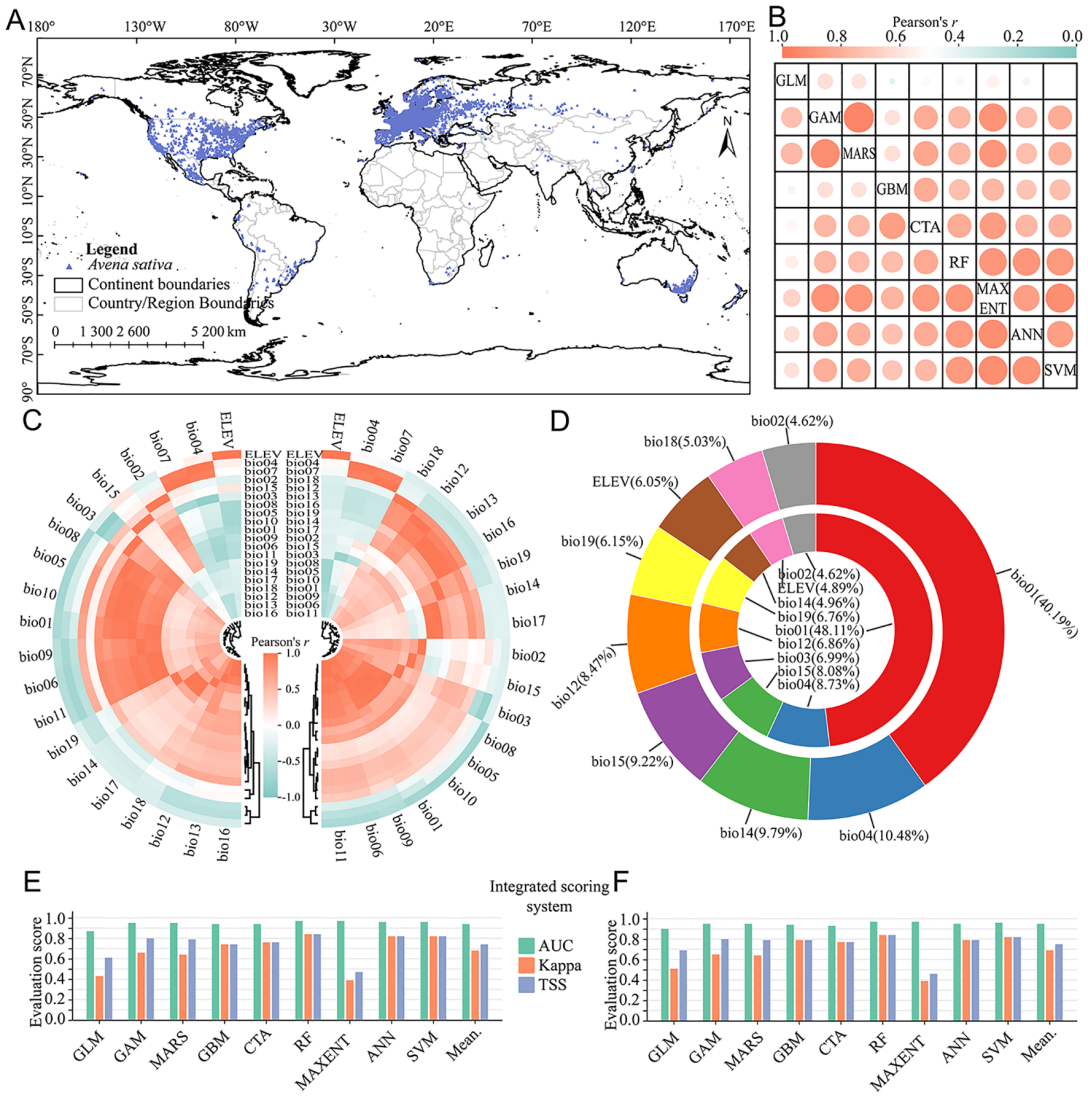
Modeling data processing and species distribution model evaluation. **(A)** Geographical distribution records of*A. sativa*; **(B)** The correlation among different algorithms in the current (upper triangles) and future (lower triangles) period; **(C)** Correlation among environmental variables in the current (left side) and future (right side) period; **(D)** Relative contribution of environmental variables in the current (outside) and future (inside) period; **(E, F)** Evaluation of integrated scoring system of different algorithms in current **(E)** and future **(F)** period. The meanings of abbreviations for environmental factors were shown in **Supplementary Table S1**.

The global effective distribution points of *A. sativa* were 6, 215 after data quality control and rarefy (**Figure 2A**), which had a wide distribution range, with record densities much higher in North America and Europe than in other continents. After correlation analysis of all environmental factors, 8 identical environmental factors were selected from current and future period, including bio01, bio02, bio04, bio12, bio14, bio15, bio18, bio19 and ELEV. The bio18 and bio03 were specifically included in data of current and future period. The correlation ranges between algorithms in current and future period were 0.39~0.95 and 0.44~0.92, respectively (**Figure 2B**), which indicated that there are significant deviations and uncertainties among different algorithms. Overall, for the modeling results in current and future period, the AUC, Kappa and TSS were 0.94, 0.68, 0.74 (**Figure 2E**) and 0.95, 0.69, 0.75 (**Figure 2F**), respectively. The evaluation results of integrated scoring system indicated that the ensemble model constructed by different algorithms can effectively simulate the potential spatial distribution pattern of *A. sativa*.

**Figure 3:**
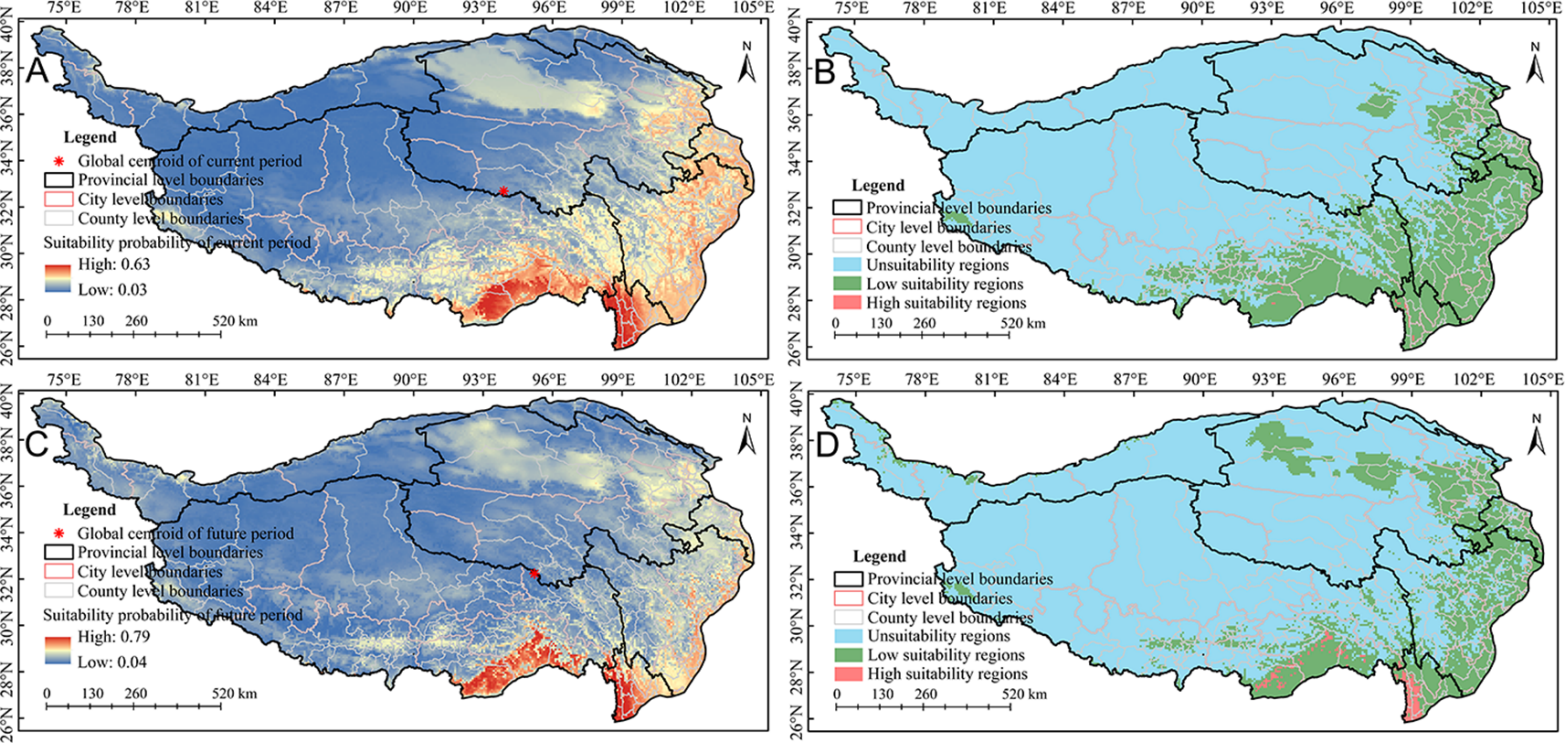
Potential spatial distribution pattern of*A. sativa*. **(A, B)** Potential suitability probability **(A)** and potential suitability pattern **(B)** in the current period; **(C, D)** Potential suitability probability **(C)** and potential suitability pattern **(D)** in the future period.

Relative contribution can be used to evaluate the influence of environmental variables upon the spatial distribution of *A. sativa*. The relative contribution analysis showed that the cumulative relative contribution of 5 environmental factors exceeded 75% (**Figure 2C**), including bio01, bio04, bio14, bio15, bio12 in current period, and bio01, bio04, bio15, bio03, bio12 in future period. Overall, the spatial distribution pattern of *A. sativa* was obviously responsive to hydrothermal environmental conditions, especially bio01. Based on the natural break classification method, the potential spatial distribution of *A. sativa* was divided into three suitability levels (**Supplementary Table S8**). The results showed that the potential suitability probability in the QTP region ranged 0.03 to 0.63, and potential spatial distribution mainly spans 26°~37°N and 88°~105°E in current period (**Figure 3A**); while the potential suitability probability ranged 0.04 to 0.79, and potential spatial distribution mainly spans 26°~39°N and 92°~105°E in future period (**Figure 3C**). The potential distribution of *A. sativa* in the current and future periods was concentrated in the southeastern part of QTP.

This study predicted an area of 2.55×10^6^ km^2^ in the QTP, with high and low suitability regions accounting for 0.08% (1.90×10^3^ km^2^) and 22.78% (5.81×10^5^ km^2^), respectively. The high and low suitability regions involve 3 and 37 municipal administrative regions respectively (**Supplementary Table S9**), among which the top three municipal administrative regions in high suitability regions were Nujiang Li Autonomous Prefecture (1.50×10^3^ km^2^), Shannan City (300 km^2^) and Diqing Tibetan Autonomous Prefecture (100 km^2^) (**Figure 3B**); The top three municipal administrative regions in low suitability regions were Garze Tibetan Autonomous Prefecture (9.12×10^4^ km^2^), Linzhi City (8.28×10^3^ km^2^) and Aba Tibetan and Qiang Autonomous Prefecture (6.72×10^3^ km^2^) (**Figure 3B**). Overall, the suitability area for *A. sativa* distribution accounts for about 22.85% of the total area in the QTP. The dynamic changes of the potential spatial distribution pattern of *A. sativa* across multiple periods were analyzed. The results showed that comparing the future period with current period, the overall suitability area had increased by -14.43%, reaching 4.98×10^5^ km^2^, among them, the high and low suitability regions increased by 917.86% (up to 1.98×10^5^ km^2^) and -17.55% (up to 4.79×10^5^ km^2^), respectively (**Figure 3D**). Overall, compared to the present, the forecasted suitability area for *A. sativa* demonstrated a general collapse, however, the high suitability regions showed an expanding trend. Furthermore, the centroid of the suitability habitat was estimated to migrate southeastward by approximately 134.66 km (**Figure 3**).

### 3.6 Prediction of introduction adaptability of *Avena sativa*

**Figure 4:**
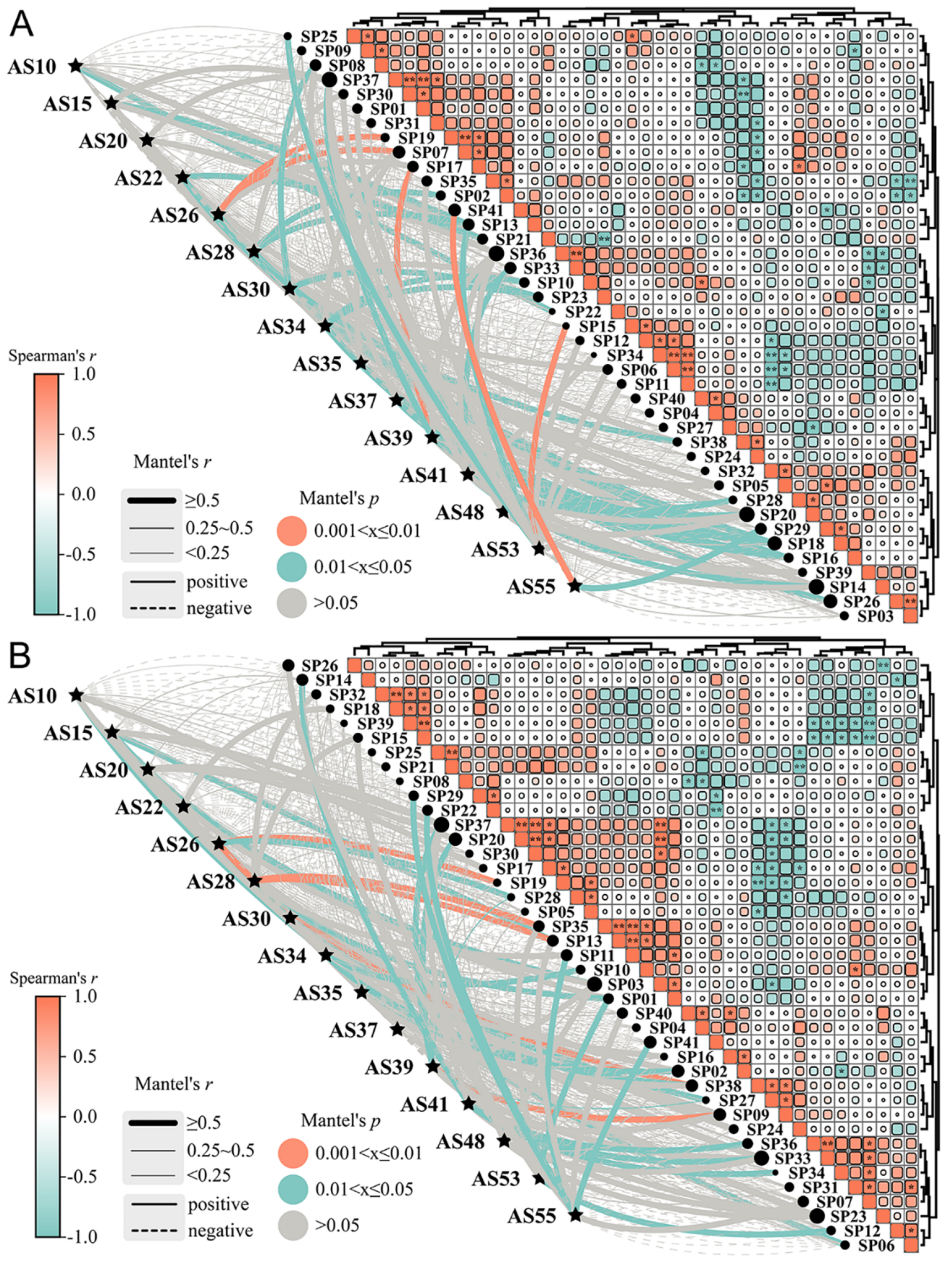
Prediction of introduction adaptability of different germplasms introductions of*A. sativa* under different periods. **(A, B)** Mantel Test analysis between tested materials and sample plots in the current **(A)** and future **(B)** period; The meanings of abbreviations for agronomic traits were shown in **Supplementary Table S3**; SP01: Chengdu City; SP02: Chongzhou City; SP03: Panzhihua City; SP04: Deyang City; SP05: Mianyang City; SP06: Ya’an City; SP07: Aba Tibetan and Qiang Autonomous Prefecture; SP08: Garze Tibetan Autonomous Prefecture; SP09: Liangshan Yi Autonomous Prefecture; SP10: Lijiang City; SP11: Dali Bai Autonomous Prefecture; SP12: Nujiang Li Autonomous Prefecture; SP13: Diqing Tibetan Autonomous Prefecture; SP14: Lasa City; SP15: Shigatse City; SP16: Changdu City; SP17: Linzhi City; SP18: Shannan City; SP19: Nagqu City; SP20: Ali region; SP21: Yongdeng County; SP22: Wuwei City; SP23: Zhangye City; SP24: Jiuquan City; SP25: Dingxi City; SP26: Longnan City; SP27: Linxia Hui Autonomous Prefecture; SP28: Haidong City; SP29: Gannan Tibetan Autonomous Prefecture; SP30: Xining City; SP31: Haibei Tibetan Autonomous Prefecture; SP32: Huangnan Tibetan Autonomous Prefecture; SP33: Hainan Tibetan Autonomous Prefecture; SP34: Guoluo Tibetan Autonomous Prefecture; SP35: Yushu Tibetan Autonomous Prefecture; SP36: Haixi Mongol and Tibetan Autonomous Prefecture; SP37: Golmud City; SP38: Bayingolin Mongolian Autonomous Prefecture; SP39: Kizilsu Kyrgyz Autonomous Prefecture; SP40: Kashgar region; SP41: Hotan City.

15 germplasms selected from different evaluation dimensions were used for introduction adaptability prediction among 41 municipal administrative regions related to the QTP. The mantel analysis of introduction adaptability showed that the spearman’s correlation ranged from -1.00 to 1.00 among all regions in the current and future periods (**Figure 4**). For example, the correlation among SP01, SP30 and SP37 was all greater than 0.8, which indicated that these regions had similar environmental conditions to adapt to *A. sativa*, while the correlation among SP33, SP36 and SP39 was all less than -0.8, which indicated that the adaptability probability of these regions was quite different. 15 germplasms had varying degrees of introduction adaptability in 23 municipal administrative regions in the current period (**Figure 4A**), with a total suitable introduction area of 7.81×10^5^ km^2^, including 1.60×10^3^ km^2^ of high suitability regions (**Supplementary Table S10**). Specifically, AS26 was highly significant positively correlated with Aba Tibetan and Qiang Autonomous Prefecture (SP07) and Nagqu City (SP19); AS39 with Linzhi City (SP17); AS53 with Shigatse City (SP15) and AS55 with Hotan City (SP41), which indicated that each of the above germplasms was suitable for planting in similar regions represented by corresponding administrative regions. It is worth noting that, considering the potential suitability distribution of *A. sativa* (**Figure 3**), AS26 was suitable for planting 85.51% of SP07 and 0.41% of SP19; AS39 was suitable for planting in 75.11% of SP17; AS53 was suitable for planting in 12.98% of SP15. However, SP41 did not contain areas suitable for introducing AS55 (**Supplementary Table S10**). Compared with the current period, 15 germplasms in the future period also had different degrees of introduction adaptability in 23 municipal administrative regions (**Figure 4B**), and the area of suitable introduction decreased by 65.62% to 2.68×10^5^ km^2^, including 1.38×10^4^ km^2^ of high suitability regions (**Supplementary Table S11**). Overall, with the passage of time, the introduction adaptability of *A. sativa* with different germplasms to different regions also changed dynamically due to climate change.

## 4 Discussion

### 4.1 Influencing factors of forage yield for *A. sativa*

Under the same field management conditions, the evaluation of forage fertility period is an important indicator for the adaptability of forage germplasm to the environment of the experimental regions (Caradus and Chapman, 2024). In this study, 19 late-maturing within 62 A*. sativa* germplasms could not effectively complete the fertility period, and were in vegetative growth, flowering and grain filling stages to varying degrees, which was similar to the research results of predecessors (Peng et al., 2018). Traditionally, it is believed that the growth time of *A. sativa* in high altitude areas is 84~159 days (Xu, 2012). The high altitude, low temperature and short frost-free period of the QTP pose a risk of frost for late-maturing crops (Suonan et al., 2024). Therefore, early and medium-maturing germplasms should be the main ones in the breeding of germplasm suitable for *A. sativa* in the QTP. The different agronomic traits of *A. sativa* germplasms had different effects on forage yield and adaptability, and this study found that the forage yield was positively affected by leaf, stem, and spike traits, while also significantly negatively affecting grain traits. Leaf was key organs for plant growth, photosynthesis and water balance (Xu, 2012). In this study, the area of flag and flag-second leaf was significantly positively correlated with forage yield, indicating that the larger the leaf area and the higher the forage yield, which may be the result of the accumulation of photosynthetic products in the leaves (Shah et al., 2015). Plant height and tiller number are often important components of forage yield, and are used to evaluate the adaptability of *A. sativa*. In this study, the plant height of *A. sativa* ranged from 102.80 to 157.80 cm, and the tiller number ranged from 11.80 to 36.80, which were significantly positively correlated with forage yield. This phenomenon means that plant height and tiller number can positively affect forage yield of *A. sativa*. The stem to leaf ratio is particularly important for evaluating palatability and forage utilization value (Lee, 2018). A smaller stem to leaf ratio and higher leaf content indicates better palatability and more suitable for feeding livestock (Mganga et al., 2021). The results of this study showed a highly significant positive correlation between stem to leaf ratio and forage yield. The germplasm AS26 with the highest stem to leaf ratio did not have the highest forage yield. The reason for this result should be that the forage yield of *A. sativa* was determined by multiple agronomic traits and environmental factors, and the genetic characteristics of germplasm were also important factors in determining forage yield (Buxton, 1996). This study found that the thousand kernel weight of *A. sativa* was negatively regulated by forage yield. Previous research also found the thousand kernel weight of *A. sativa* was negatively correlated with forage yield, spike length and plant height (Wu et al., 2023), and crops will store more proteins before flowering, and then these proteins will be hydrolyzed to form amino acids for seed filling, leading to a decrease in plant biomass (Zhou et al., 2016). The biomass of increased will compete for the nutrition delivered to grains, it is necessary to reasonably consider the trade-off between maximum biomass and high crude protein content. Therefore, for specific breeding objectives, it is necessary to simultaneously pay attention to the interaction and development of multiple traits in the breeding process of *A. sativa*.

### 4.2 Adaptability of forage yields across different *A. sativa* germplasms in the QTP region

Forage yield, including hay and fresh grass, is an important indicator to measure the field production performance of *A. sativa*, and it is also a visual display of adaptability and stability under different environmental conditions (Desheva and Valchinova, 2024). In this study, there were great differences in forage yield among different germplasms of *A. sativa*. Based on the hay grass yield data, the clustering results showed that the class composed of AS55 and AS48 had the highest yield, which can be used as an excellent parent for breeding with high hay grass yield; the class composed of AS39, AS28, AS41, AS22, AS20, AS15 and AS34 had high hay grass yield and could be used as forage germplasm for production. Based on the fresh grass yield data, the clustering results indicated that the class composed only of AS55 had the highest yield and was suitable as an excellent parent for breeding, while the class composed of AS48, AS41, AS22 and AS39 had higher grass yield and was suitable for producing. The comprehensive evaluation of different agronomic traits also indicated that the class composed of AS55 had the best comprehensive production performance in the field, followed by the class composed of 14 germplasms such as AS39, AS48, AS28, AS41, AS20, AS15, AS22, AS34, AS37, AS30, AS35, AS53, AS26 and AS10.

Environment has an important influence on the distribution, diffusion and adaptability of species, which directly or indirectly affects the distribution of species through the regulation of climate or geographical isolation (Zhang et al., 2018). In this study, the identified key environmental factors affecting the spatial distribution of *A. sativa* in different periods were obviously responsive to hydrothermal environmental conditions, which was consistent with the viewpoint that abiotic factors, mainly climate factors, dominated the species distribution pattern on a large scale (Soberon and Peterson, 2005). Temperature and precipitation are the main climatic factors affecting pasture growth (Li et al., 2018). *A. sativa* is mainly cultivated in arid or semi-arid areas, and precipitation is the main influencing factor of yield formation (Zhang et al., 2019). Mean annual air temperature (bio01) is an indicator reflecting the overall heat conditions during the year. As a crop that prefers cold and fears high temperatures (Sorrells and Simmons, 1992), this study predicted that compared to precipitation, temperature had a greater impact on the potential spatial distribution of *A. sativa*. Suitable annual average temperature is helpful for the plant growth. Larger temperature differences contribute to the accumulation of dry matter, which in turn affects the duration, phenology, yield and quality of *A. sativa* in the field. Temperature has an important influence on the productivity of alpine grassland plants, but the continuous rise of temperature may have a negative impact on alpine grasslands (Zhou et al., 2019). The potential suitable planting areas for *A. sativa* in different periods were concentrated in the southeastern part of the QTP. With the climate warming and drought intensification in the future, the potential suitability regions of *A. sativa* generally show a collapse trend, but the high suitability regions show an obvious expansion trend, which indicates that the suitable planting space of *A. sativa* has potential. Habitat similarity provides a potential perspective for exploring the adaptive responses of plants in different regions (Kerr and Packer, 1997). The results showed that 15 A*. sativa* germplasms had different degree of adaptability in 23 municipal administrative regions of the QTP. However, with the passage of time and climate change, the adaptability of different germplasms to different regions also underwent dynamic changes. This indicates that when considering the adaptability of different germplasms to the environment, it is important to dynamically recognize the growth characteristics and agronomic response of *A. sativa* to the habitat background.

## Data Availability

The original contributions presented in the study are included in the article/**Supplementary Material**. Further inquiries can be directed to the corresponding author.

## References

[B1] Brown J. L. Bennett J. R. French C. M. (2017). SDMtoolbox 2.0: the next generation Python-based GIS toolkit for landscape genetic, biogeographic and species distribution model analyses. PeerJ 5, e4095. doi: 10.7717/peerj.4095, PMID: 29230356 PMC5721907

[B2] Buxton D. R. (1996). Quality-related characteristics of forages as influenced by plant environment and agronomic factors. Anim. Feed Sci. Tech. 159, 37–49. doi: 10.1016/0377-8401(95)00885-3

[B3] Cao J. Xu X. Deo R. C. Holden N. M. Adamowski J. F. Gong Y.. (2018). Multi-household grazing management pattern maintains better soil fertility. Agron. Sustain. Dev. 38, 6. doi: 10.1007/s13593-017-0482-2

[B4] Caradus J. R. Chapman D. F. (2024). Evaluating pasture forage plant breeding achievements: a review. New Zeal. J. Agr. Res. 8, 1–75. doi: 10.1080/00288233.2024.2395370

[B5] Desheva G. Valchinova E. (2024). Evaluation of yield stability and adaptability of oat genotypes (*Avena sativa* L.). Poljoprivreda 30, 3–12. doi: 10.18047/poljo.30.1.1

[B6] Dong S. Wen L. Liu S. Zhang X. Lassoie J. P. Yi S.. (2011). Vulnerability of worldwide pastoralism to global changes and interdisciplinary strategies for sustainable pastoralism. Ecol. Soc 16, 10. doi: 10.5751/ES-04093-160210

[B7] Jägermeyr J. Müller C. Ruane A. C. Elliott J. Balkovic J. Castillo O.. (2021). Climate impacts on global agriculture emerge earlier in new generation of climate and crop models. Nat. Food 2, 873–885. doi: 10.1038/s43016-021-00400-y, PMID: 37117503

[B8] Kebede G. Worku W. Feyissa F. Jifar H. (2023). Genotype by environment interaction and stability analysis for selection of superior fodder yield performing oat (*Avena sativa* L.) genotypes using GGE biplot in Ethiopia. Ecol. Genet. Genomics 28, 100192. doi: 10.1016/j.egg.2023.100192

[B9] Kerr J. T. Packer L. (1997). Habitat heterogeneity as a determinant of mammal species richness in high-energy regions. Nature 385, 252–254. doi: 10.1038/385252a0

[B10] Lee M. A. (2018). A global comparison of the nutritive values of forage plants grown in contrasting environments. J. Plant Res. 131, 641–654. doi: 10.1007/s10265-018-1024-y, PMID: 29550895 PMC6015622

[B11] Li R. Zhang Z. Tang W. Huang Y. Coulter J. A. Nan Z. (2020). Common vetch cultivars improve yield of oat row intercropping on the Qinghai-Tibetan plateau by optimizing photosynthetic performance. Eur. J. Agron. 117, 126088. doi: 10.1016/j.eja.2020.126088

[B12] Li X. Zhao F. Lin W. (2018). Impacts of climate change on growth and development of pasture: A Review. Chin. Agricult. Sci. Bull. 34, 145–152. doi: 10.11924/j.issn.1000-6850.casb18040099

[B13] Liang G. L. Qin Y. Wei X. X. Liu Y. C. Liu Y. Liu W. H. (2018). Evaluation on productivity and quality of oat strain I-D in the alpine regions of the Qinghai-Tibetan Plateau. Acta Agrestia Sin. 26, 917–927. doi: 10.11733/j.issn.1007-0435.2018.04.017

[B14] Liu W. Li B. Yuan Y. Li Y. Jiang Y. Li R.. (2023). Artificial grassland mapping using artificial grassland detection index of vegetation growth in the Three-River Headwaters region. Ecol. Indic. 154, 110869. doi: 10.1016/j.ecolind.2023.110869

[B15] Lu H. H. Zheng Y. Y. Qiu Y. S. Tang L. B. Zhao Y. C. Xie W. G. (2025). Comprehensive prediction of potential spatiotemporal distribution patterns, priority planting regions, and introduction adaptability of *Elymus sibiricus* in the Chinese region. Front. Plant Sci. 15, 1470653. doi: 10.3389/fpls.2024.1470653, PMID: 39845496 PMC11751619

[B16] Mganga K. Z. Ndathi A. J. N. Wambua S. M. Bosma L. Kaindi E. M. Kioko T.. (2021). Forage value of vegetative leaf and stem biomass fractions of selected grasses indigenous to African rangelands. Anim. Prod. Sci. 61, 1476–1483. doi: 10.1071/AN19597

[B17] Miller J. (2010). Species distribution modeling. Geog. Compass 4, 490–509. doi: 10.1111/j.1749-8198.2010.00351.x

[B18] Peng X. Q. Zhou Q. P. Liu W. H. Wei X. X. Tian L. H. Chen Y. J.. (2018). A comparative analysis of growth characteristics of six oat cultivars in the north-western Sichuan alpine region. Pratacult. Sci. 12, 1208–1217. doi: 10.11829/j.issn.1001-0629.2017-0661

[B19] Ren C. Y. Liang G. L. Liu W. H. Liu K. Q. Duan J. L. (2023). Screening and adaptability evaluation of early maturing oats in alpine regions of the Qinghai-Tibetan Plateau. Acta Pratacult. Sin. 32, 116–129. doi: 10.11686/cyxb2022414

[B20] Riahi K. van Vuuren D. P. Kriegler E. Edmonds J. O’Neill B. C. Fujimori S.. (2017). The Shared Socioeconomic Pathways and their energy, land use, and greenhouse gas emissions implications: An overview. Global Environ. Change 42, 153–168. doi: 10.1016/j.gloenvcha.2016.05.009

[B21] Sánchez-Martín J. Rubiales D. Flores F. Emeran A. A. Shtaya M. J. Y. Sillero J. C.. (2014). Adaptation of oat (*Avena sativa*) cultivars to autumn sowings in Mediterranean environments. Field Crop Res. 156, 111–122. doi: 10.1016/j.fcr.2013.10.018

[B22] Schmitt S. Pouteau R. Justeau D. Boissieu F. D. Birnbaum P. (2017). ssdm: An r package to predict distribution of species richness and composition based on stacked species distribution models. Methods Ecol. Evol. 8, 1795–1803. doi: 10.1111/2041-210X.12841

[B23] Shah S. A. S. Akhtar L. H. Minhas R. Bukhari S. M. S. Ghani A. Anjum M. H. (2015). Evaluation of different oat (*Avena sativa* L.) varieties for forage yield and related characteristics. Sci. Lett. 3, 13–16.

[B24] Soberon J. Peterson A. T. (2005). Interpretation of models of fundamental ecological niches and species’ distributional areas. Biodivers. Inform 2, 1–10. doi: 10.17161/bi.v2i0.4

[B25] Sorrells M. E. Simmons S. R. (1992). Influence of environment on the development and adaptation of oat. Oat Sci. Technol. 33, 115–163. doi: 10.2134/agronmonogr33.c5

[B26] Steinitz O. Heller J. Tsoar A. Rotem D. Kadmon R. (2006). Environment, dispersal and patterns of species similarity. J. Biogeogr. 33, 1044–1054. doi: 10.1111/j.1365-2699.2006.01473.x

[B27] Suonan J. Lü W. Classen A. T. Wang W. La B. Lu X.. (2024). Alpine plants exhibited deep supercooling upon exposed to episodic frost events during the growing season on the Qinghai-Tibet Plateau. J. Plant Ecol. 17, rtae034. doi: 10.1093/jpe/rtae034

[B28] Wang Q. Li Z. J. Li J. Zhou B. W. (2019). Evaluation of agronomic and forage quality traits of a range of oat cultivars. Acta Pratacult. Sin. 28, 149–158. doi: 10.11686/cyxb2019040

[B29] Watts M. E. Stewart R. R. Martin T. G. Klein C. J. Carwardine J. Possingham H. P. (2017). Systematic conservation planning with Marxan. Learn. Landscape Ecol. 5, 211–227. doi: 10.1007/978-1-4939-6374-4_13

[B30] Wu J. Y. Ma L. Wu X. M. Shi Y. H. Chen F. E. Wang X. M.. (2023). Study on phenotypic genetic diversity of 180 feed Oat germplasms. Acta Agrestia Sin. 31, 1501–1510. doi: 10.11733/j.issn.1007-0435.2023.05.025

[B31] Xu C. L. (2012). A study on growth characteristics of different cultivars of oat (*Avena sativa*) in alpine region. Acta Pratacult. Sin. 21, 280–285.

[B32] Ye X. L. Gan Z. Wan Y. Xiang D. B. Wu X. Y. Wu Q.. (2023). Advances and perspectives in forage oat breeding. Acta Pratacult. Sin. 32, 160–177. doi: 10.11686/cyxb2022263

[B33] Zhang Y. Duan Y. Nie J. Yang J. Ren J. van der Werf W.. (2019). A lack of complementarity for water acquisition limits yield advantage of oats/vetch intercropping in a semi-arid condition. Agr. Water Manage. 225, 105778. doi: 10.1016/j.agwat.2019.105778

[B34] Zhang J. Guo G. Chen L. Li J. Yuan X. Yu C.. (2015). Effect of applying lactic acid bacteria and propionic acid on fermentation quality and aerobic stability of oats-common vetch mixed silage on the Tibetan plateau. Anim. Sci. J. 86, 595–602. doi: 10.1111/asj.12340, PMID: 25494579

[B35] Zhang F. L. Wang X. Zhang J. (2018). Biodiversity information resources. II. Environmental data. Biodivers. Sci. 26, 53–65. doi: 10.17520/biods.2017189

[B36] Zhou B. Serret M. D. Elazab A. Pie J. B. Araus J. L. Aranjuelo L.. (2016). Wheat ear carbon assimilation and nitrogen remobilization contribute significantly to grain yield. J. Integr. Plant Biol. 58, 914–926. doi: 10.1111/jipb.12478, PMID: 26990448

[B37] Zhou P. P. Yan H. H. Peng Y. Y. (2019). Hexaploid ancestor of cultivated hexaploid oats inferred from high throughput GBS-SNP markers. Acta Agronom. Sin. 45, 1604–1612. doi: 10.3724/SP.J.1006.2019.81091

[B38] Zhou H. Yang X. Zhou C. Shao X. Shi Z. Li H.. (2023). Alpine grassland degradation and its restoration in the Qinghai–Tibet plateau. Grasses 2, 31–46. doi: 10.3390/grasses2010004

[B39] Zizka A. Silvestro D. Andermann T. Azevedo J. Ritter C. D. Edler D.. (2019). CoordinateCleaner: Standardized cleaning of occurrence records from biological collection databases. Methods Ecol. Evol. 10, 744–751. doi: 10.1111/2041-210X.13152

