# Supplementary material for: Evaluation of forage production performance and prediction of introduction adaptability for different Avena sativa germplasms in the southeast edge of the Qinghai-Tibet plateau
Source: Front Plant Sci. 2025 Aug 13;16:1564278. doi: 10.3389/fpls.2025.1564278 (PMC12380873; doi:10.3389/fpls.2025.1564278)
Supplement: Supplementary file 1 [file Table1.docx]

Supplementary Material

# Supplementary Figures


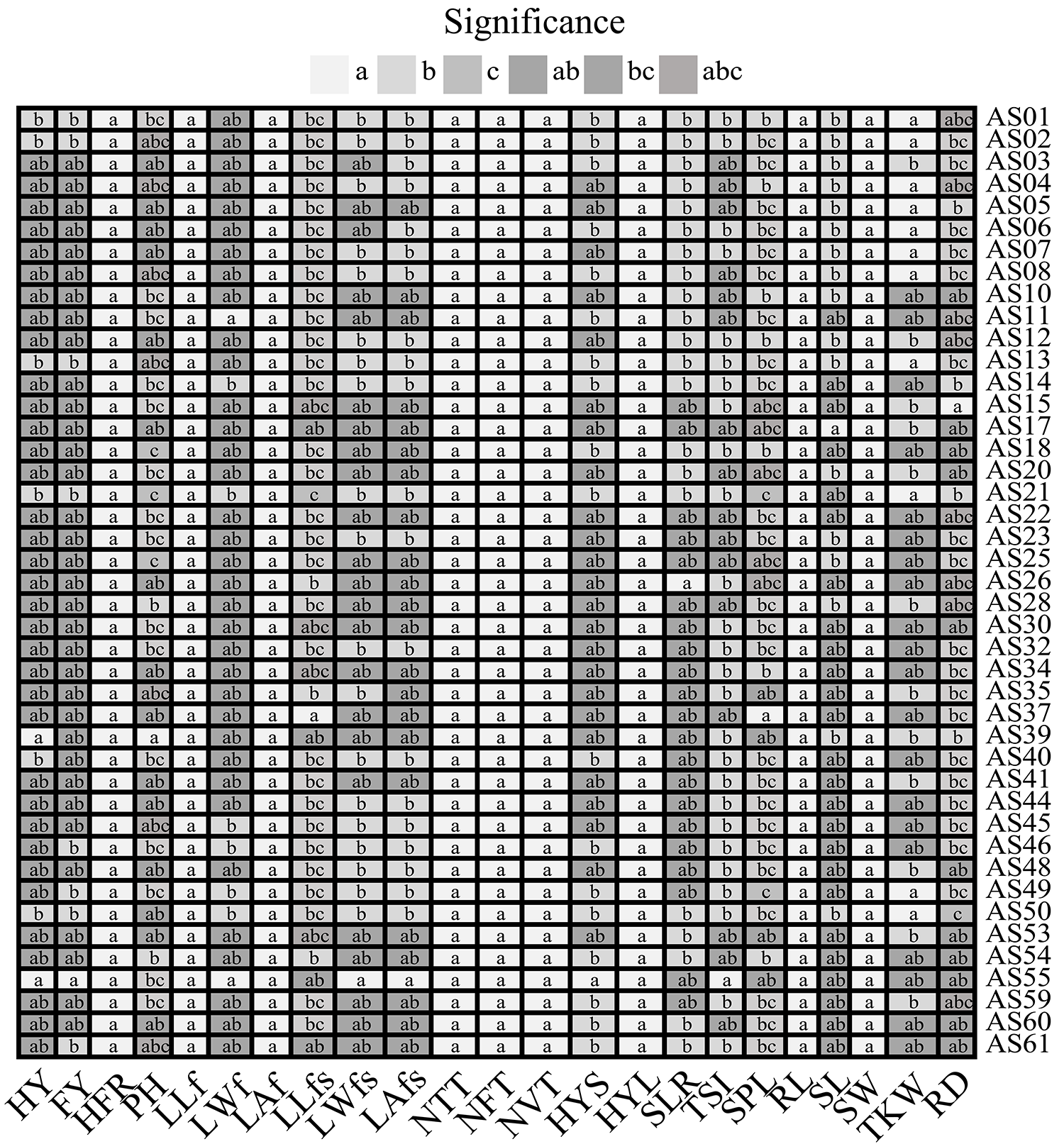


**Supplementary Figure 1.** **Post hoc multiple tests between different agronomic traits across different *A. sativa* germplasm.**
